# Heat Shock Factor Genes of Tall Fescue and Perennial Ryegrass in Response to Temperature Stress by RNA-Seq Analysis

**DOI:** 10.3389/fpls.2015.01226

**Published:** 2016-01-11

**Authors:** Yan Wang, Ya Dai, Xiang Tao, Jia-Zhen Wang, Hai-Yang Cheng, Hong Yang, Xin-Rong Ma

**Affiliations:** ^1^Chengdu Institute of Biology, Chinese Academy of SciencesChengdu, China; ^2^University of Chinese Academy of SciencesBeijing, China; ^3^School of Life Sciences, Zunyi Normal CollegeZunyi, China

**Keywords:** heat shock factors (Hsfs), cold stress, heat stress, RNA-Seq, expression analysis, phylogenetic analysis, tall fescue, perennial ryegrass

## Abstract

Heat shock factors (Hsfs) are important regulators of stress-response in plants. However, our understanding of *Hsf* genes and their responses to temperature stresses in two *Pooideae* cool-season grasses, *Festuca arundinacea*, and *Lolium perenne*, is limited. Here we conducted comparative transcriptome analyses of plant leaves exposed to heat or cold stress for 10 h. Approximately, 30% and 25% of the genes expressed in the two species showed significant changes under heat and cold stress, respectively, including subsets of *Hsfs* and their target genes. We uncovered 74 *Hsf*s in *F. arundinacea* and 52 *Hsf*s in *L. perenne*, and categorized these genes into three subfamilies, *HsfA, HsfB*, and *HsfC* based on protein sequence homology to known Hsf members in model organisms. The *Hsfs* showed a strong response to heat and/or cold stress. The expression of *HsfA*s was elevated under heat stress, especially in class *HsfA2*, which exhibited the most dramatic responses. *HsfBs* were upregulated by the both temperature conditions, and *HsfCs* mainly showed an increase in expression under cold stress. The target genes of *Hsfs*, such as heat shock protein (*HSP*), ascorbate peroxidase (*APX*), inositol-3-phosphate synthase (*IPS*), and galactinol synthase (*GOLS1*), showed strong and unique responses to different stressors. We comprehensively detected *Hsfs* and their target genes in *F. arundinacea* and *L. perenne*, providing a foundation for future gene function studies and genetic engineering to improve stress tolerance in grasses and other crops.

## Introduction

Climate change has resulted in harmful effects on plant growth (Hasanuzzaman et al., 2013; Miura and Furumoto, 2013). The environment in which a plant thrives is species-dependent, and as a result, each species has common and unique stress-response mechanisms (Yeh et al., 2012). Tall fescue (*Festuca arundinacea*) and perennial ryegrass (*Lolium perenne*) are two major species of forage and turf grasses, which are extensively planted in warm temperate to subtropical regions around the world because they are easy to plant, have better tolerance to abiotic stresses, and require low maintenance (Raeside et al., 2012; Huang et al., 2014). However, the regions that use tall fescue and perennial ryegrass experience high temperature over 38°C in the summer, and low temperature below −10°C in the winter almost every year, which greatly exceeds the growth temperature of these cool-season grasses, and reduces the forage yield and lawn quality. Accordingly, improving tall fescue and perennial ryegrass adaptability to high and/or low temperature can increase forage yield and lawn quality in regions where it is heavily planted.

Plants cope with adverse environmental changes by altering expression of stress-related genes (Krasensky and Jonak, 2012). Heat shock proteins (HSPs), which include HSP70 (DnaK), the chaperonins (GroEL and HSP60), HSP90, HSP100 (Clp), and the small HSP (sHSP) families (Wang et al., 2004), are important molecular chaperones, and their expression is induced by environmental stressors such as heat, waterlog, salinity, osmosis, cold, and oxidation (Timperio et al., 2008; Scharf et al., 2012; Hu et al., 2015). HSPs prevent aggregation of denatured proteins, and assist in folding of nascent polypeptides and refolding denatured proteins, as well as to re-solubilize aggregated denatured proteins (Wang et al., 2004; Timperio et al., 2008; Waters, 2013).

Transcription of *HSP*s is regulated by Heat shock factors (Hsfs) (Miller and Mittler, 2006; Scharf et al., 2012; Xue et al., 2014), which are transcriptional activators of heat shock genes. More recently, it has been shown that Hsfs not only regulate the expression of *HSP* genes, but also other *Hsf* genes, and genes encoding stress-induced metabolic enzymes, such as ascorbate peroxidase 2 (*APX2*), inositol-3-phosphate synthase 2 (*IPS2*), and galactinol synthase 1 (*GOLS1*) (Miller and Mittler, 2006; Scharf et al., 2012). Hsfs form homotrimers that binds to highly conserved heat shock elements (HSE; a palindromic motif of nGAAn) in the promoter region of target genes (Peteranderl et al., 1999). The plant *Hsf* gene family is divided into three subfamilies: *HsfA, HsfB*, and *HsfC*. Each subfamily is further divided into to different classes. All *Hsf* genes contain a DNA-binding domain (DBD), an oligomerization domain (HR-A and HR-B), and a nuclear localization sequence (NLS) (Scharf et al., 2012). Hsf A proteins contain an activator peptide motif (AHA motif) in the C-terminal domains (Döring et al., 2000; Kotak et al., 2004), and Hsf B proteins, except for Hsf B5, contain the tetrapeptide LFGV, which is suggested to be a repressor motif (Czarnecka-Verner et al., 2004; Ikeda and Ohme-Takagi, 2009).

*Hsf* family genes show high copy number across different model organisms. The smallest *Hsf* family that has been observed to date comprises of 18 or 19 *Hsfs* found in ricinus, vitis, citrus, and carica (Scharf et al., 2012). The largest number of *Hsfs* have been observed in wheat, which has 56 *Hsf* genes (*Triticum aestivum*) (Xue et al., 2014). Most *Hsfs* are present in both eudicots and monocots. However, Hsf B3 and Hsf B5 are confined to eudicots. The Hsf C group shows a substantially higher level of complexity in monocots, containing monocot-specific types C1b, C2a, and C2b.

*Hsf* A subfamily genes act as activators, and have been extensively characterized in various model plant species. All genes under the Hsf A1, A2, A3, and A4 category are involved in thermotolerance in plants (Doerks et al., 2002; Baniwal et al., 2007; Zhang et al., 2009; Scharf et al., 2012). Of the Hsf A1 members, HsfA1a functions as the main and irreplaceable regulator for acquired thermotolerance in tomato (Nishizawa-Yokoi et al., 2011); however, this is not the case in *Arabidopsis thaliana* (Lohmann et al., 2004). Transgenic *A. thaliana* plants overexpressing *AtHsfA2* have increased higher thermotolerance, as well as an increased resistance to salt, osmosis, and oxidative stress (Ogawa et al., 2007; Zhang et al., 2009). *Hsf* A3 also participates in drought stress signaling that is dependent on DREB2A (dehydration-responsive element binding protein 2A) in *Arabidopsis* (Yoshida et al., 2008; Chen et al., 2010). Moreover, *Hsf* A9 regulates HSP expression during embryogenesis and seed maturation in sunflower (*Helianthus annuus, Ha*) and *A. thaliana* (Almoguera et al., 2002; Kotak et al., 2007), and is also involved in the hormonal control networks regulated by abscisic acid (ABA) and auxins (Carranco et al., 2010).

In contrast to the subfamily Hsf A, the subfamilies Hsf B and C have been less characterized. Most Hsf B members do not show activator function (Ikeda and Ohme-Takagi, 2009). However, Hsf B1 and Hsf A1a form a synergistic coactivator that regulates the different stages of tomato heat stress (HS) response (Bharti et al., 2004). Moreover, Hsf B1 cooperates with other transcriptional activators that control and/or restore expression of housekeeping genes during HS (Scharf et al., 2012). To date, the role of subfamily C Hsfs remains elusive.

Given the role *Hsf* family genes play in temperature-response in various plant species, our research group has been highly interested in determining whether *Hsf* family genes play a role in regulating heat and cold tolerance in grasses. However, there is no clear classification of *Hsf* genes and whether their expression changes under stress-response for tall fescue and perennial ryegrass. A comprehensive analysis of gene expression in the two species exposed to high or low temperature stress may allow for an improved understanding of their adaptability to temperature stress, and identify novel stress tolerance genes. Transcriptome analyses of tall fescue revealed that the heat-tolerant cultivar activated more genes compared to the heat-sensitive cultivar under heat stress, and the genes involving in sHSP, cell division and cell cycle genes showed increased expression in both cultivars (Hu et al., 2014). Previous studies have generated a single nucleotide polymorphism (SNP) library for perennial rygrass (Studer et al., 2012), and developed a reference transcriptome for multiple tissues from a highly inbred perennial ryegrass line (Farrell et al., 2014).

Tall fescue and perennial ryegrass belong to the subfamily *Pooideae*. The two species are likely closely related, as interspecific crosses occur naturally in the wild, and are employed in genetic breeding (Humphreys and Thomas, 1993; Yamada et al., 2005). To build a better reference for grass plants, we performed our experiments on the two species (population sample) instead of biological replicates of the same species.

In the present study, we generated transcriptomes of tall fescue and perennial ryegrass in response to high and low temperatures, and identified the genes closely related to temperature stress. We focused on *Hsf* genes in the two species, and classified their family members, predicted function, and analyzed the expression profile of *Hsfs* and their target genes under heat or cold stress. Our results could help elucidate the mechanism underlying the adaptability of tall fescue and perennial grass to temperature stress.

## Materials and methods

### Plant materials and temperature stress treatment

Tall fescue (*Festuca arundinacea* cv. Barlexas) and perennial ryegrass (*Lolium perenne* cv. Yatsyn) seeds were sowed in plastic pots (14 cm diameter, 25 cm length) filled with organic loam in March 2014, and seedlings were grown in a greenhouse at 18–25°C for 1 month in Chengdu (30.67°N, 104.06°E), Sichuan Province, China. The plants were clipped to a height of 8 cm, and watered by hand every 2 days. Prior to the experiment, the plants were transferred to a growth chamber set to a temperature and light cycle of 26/18°C (14 h day/10 h night) (Cool Daylight, 3070 Lm 85 Lm/W, Philips, Thailand), at relative humidity of 70%, and an irradiance of 200 mol·m^−2^s^−1^ (LI-6400/XT photometer, Li-Cor Inc., Lincoln, NE, USA). After 2 weeks, the samples from each species were divided into three parts and subjected to heat stress (HS) at 40°C, cold stress (CS) at −10°C, or maintained at the control temperature (22°C) for 10 h, with the same humidity and light conditions as above. After then, randomly selected leaves from at least 100 plants in each group were pooled, snap-frozen, and stored in liquid nitrogen. These samples were labeled FaCK (tall fescue in control temperature), FaHeat (tall fescue under HS), FaCold (tall fescue under CS), LpCK (perennial ryegrass in control temperature), LpHeat (perennial ryegrass under HS), and LpCold (perennial ryegrass under CS). Three independent sample pools from each treatment seedlings were collected by the same sampling method, snap-frozen immediately, and preserved in liquid nitrogen until use.

### Illumina deep sequencing

Total RNA samples were prepared using Trizol™ reagent (Invitrogen, Carlsbad, CA, USA), and the mRNA library was constructed using the Truseq™ RNA Sample Prep Kit (Illumina, San Diego, CA, USA) following the manufacturer's instructions. Samples were then sequenced on Illumina HiSeq™ 2000 (Illumina) in BGI (Shenzhen, China), and each sample yielded more than 4 Gb of data.

### *De novo* assembly and functional annotation

The raw reads from RNA-Seq were filtered to increase the quality of the data using the internal filter_fq software of BGI (Shenzhen, China). *De novo* assembly of the transcriptome of tall fescue and perennial ryegrass were conducted using Trinity (release-20130225) (http://trinityrnaseq.sourceforge.net/) under default parameters (Grabherr et al., 2011). The quality of the assembly was determined using length distribution, N50 number, average length, max length, and contig number at different length intervals. The assembled unigene sequences were aligned to protein databases including NR (release-20130408), Swiss-Prot (release-2013_03), the Kyoto Encyclopedia of Genes and Genomes (KEGG, Release 63.0), Cluster of Orthologous Groups of proteins (COG) (release-20090331) by blastx (*e* < 0.00001), nucleotide database NT (release-20130408) by blastn (*e* < 0.00001) (http://blast.ncbi.nlm.nih.gov/Blast.cgi), and the Gene Ontology (GO) (http://www.blast2go.com/b2ghome) (Conesa et al., 2005). The resulting unigenes were functionally annotated, and assigned the proteins with the highest sequence similarity. GO enrichment analysis was performed by using the GO-TermFinder (v0.86) software (http://search.cpan.org/dist/GO-TermFinder/). All RNA-Seq reads were deposited to the Sequence Read Archive database (http://www.ncbi.nlm.nih.gov/Traces/sra/) under the accession numbers SRP059410 (tall fescue) and SRP059405 (perennial ryegrass).

### Differential expression analysis

Unigene expression was calculated using the fragments per kilobase of exon model per million (FPKM) by the SOAP software (http://soap.genomics.org.cn/soapaligner.html) (Mortazavi et al., 2008). Differential expression levels were calculated according to the FPKM of each transcript between libraries (Audic and Claverie, 1997). Significant differences in transcript expression levels were determined by setting the thresholds for false discovery rate as “(FDR) < 0.001” and “|log_2_ Ratio| ≥ 1,” where ratio indicates the fold-change of FPKM values for the treatment and control samples. Venn diagrams for expression differences were generated online (http://bioinfogp.cnb.csic.es/tools/venny/index.html), and heatmaps were created by using the HemI 1.0 software (Deng et al., 2014).

### Classification of Hsf proteins

Hsf members were determined using the Hsf proteins from *Brachypodium distachyon* and *A. thaliana* from the HSF database (www.cibiv.at/services/hsf) as references (Scharf et al., 2012). The phylogenetic tree of Hsf proteins with DNA-binding domain was generated using ClustalW, as well as using MEGA 6.0's un-rooted neighbor-joining method with pairwise deletion and poisson correction (Tamura et al., 2013). The level of statistical support for each tree node was determined using bootstrap analysis with 1000 replicates. Partial Hsf proteins lacking or having incomplete DNA-binding domain that were recovered in the transcriptome analysis were classified based on the highest sequence homology to full-length Hsf proteins in the NCBI database, but were excluded from phylogenetic analysis.

### Quantitative real time-polymerase chain reaction (qRT-PCR) verification

cDNA was prepared from the purified RNA samples using the PrimeScript™ RT reagent kit with gDNA Eraser (Takara, Dalian, China). Gene-specific qRT-PCR primers (18–20 bp) were designed using the Premier 5.0 software (Premier Biosoft International, Palo Alto, CA). qRT-PCR was performed using AceQ SYBR Green Master Mix (Vazyme Biotech Co., Ltd, Nanjing, China) with a FTC3000 Real-Time PCR System (Funglyn, Canada). The qPCR conditions were as follows: 5 min at 95°C, 45 cycles of denaturation at 95°C for 10 s, annealing at 50–58°C (according to the Tm of primers) for 30 s, and extension at 68°C for 40 s. And all real-time reactions were performed in three biological replicates and three technical replicates, and a melting curve was used to check amplification specificity. Each gene's relative expression level was calculated based on the comparative threshold cycle Ct(2^−ΔΔ*Ct*^) method (Livak and Schmittgen, 2001) using tall fescue and perennial ryegrass β-actin 7 gene as the internal reference. The software SPSS 11.5 was used for statistical analysis.

## Results

### Global description of transcriptomes of tall fescue and perennial ryegrass

To identify genes that are differentially expressed in tall fescue and perennial ryegrass under temperature-stress, we subjected plants to heat (40°C), cold (−10°C), and control (22°C) temperatures, and collected RNA for *de novo* transcriptome assembly. After *de novo* assembly, we identified 97,565 unigenes representing a total length of 68,116,614 nt in the transcriptome of pooled tall fescue leaves in response to heat, cold, and control temperatures. The average length of the unigenes was 698 nt, with a N50 number of 1064 nt. Of these, we annotated 57,156, 55,389, 35,792, 34,063, 21,157, and 38,454 of the unigenes to the NR, NT, Swiss-Prot, KEGG, COG, and GO databases, respectively, thereby generating a total of 63,607 functionally annotated unigenes (Supplementary Table 1-S1). We obtained 62,399 coding region sequences (CDS) by translating all sequences in the transcriptome into amino sequences using the standard codon table. The CDS sequences were then aligned to protein databases in the priority order of NR, Swiss-Prot, KEGG, and COG; this resulted in 56,141 unigenes, with 6258 sequences that did not align to any database.

We also generated a *de novo* transcriptome of the perennial ryegrass leaf in response to heat, cold, and control temperatures, which resulted in a total of 73,125 unigenes. The total length of the unigenes was 52,879,856 nt, with an average length of 723 nt, and a N50 of 1133 nt. We generated 51,062 annotated unigenes by annotating 46,858, 45,050, 31,051, 29,644, 19,616, and 32,561 unigenes using the NR, NT, Swiss-Prot, KEGG, COG, and GO databases, respectively (Supplementary Table 2-S1). We then conducted a protein coding region prediction analysis, and identified a total of 51,200 CDSs, which were then mapped on to protein databases. We assigned 46,363 CDSs to a protein identity, whereas 4837 were unmapped.

The unigenes in the tall fescue or perennial ryegrass were classified into 55 GO functional groups, including 22 biological process groups, 17 cellular component groups, and 16 molecular function groups (Supplementary Figures 1A,B). KEGG analysis of the unigenes resulted in 128 pathways (Supplementary Tables 1-S2, 2-S2), and COG annotation analysis of the unigenes resulted in 25 functional categories (Supplementary Figures 2A,B). These results were consistent for both species.

### Differentially expressed genes under temperature stress

**Figure 1:**
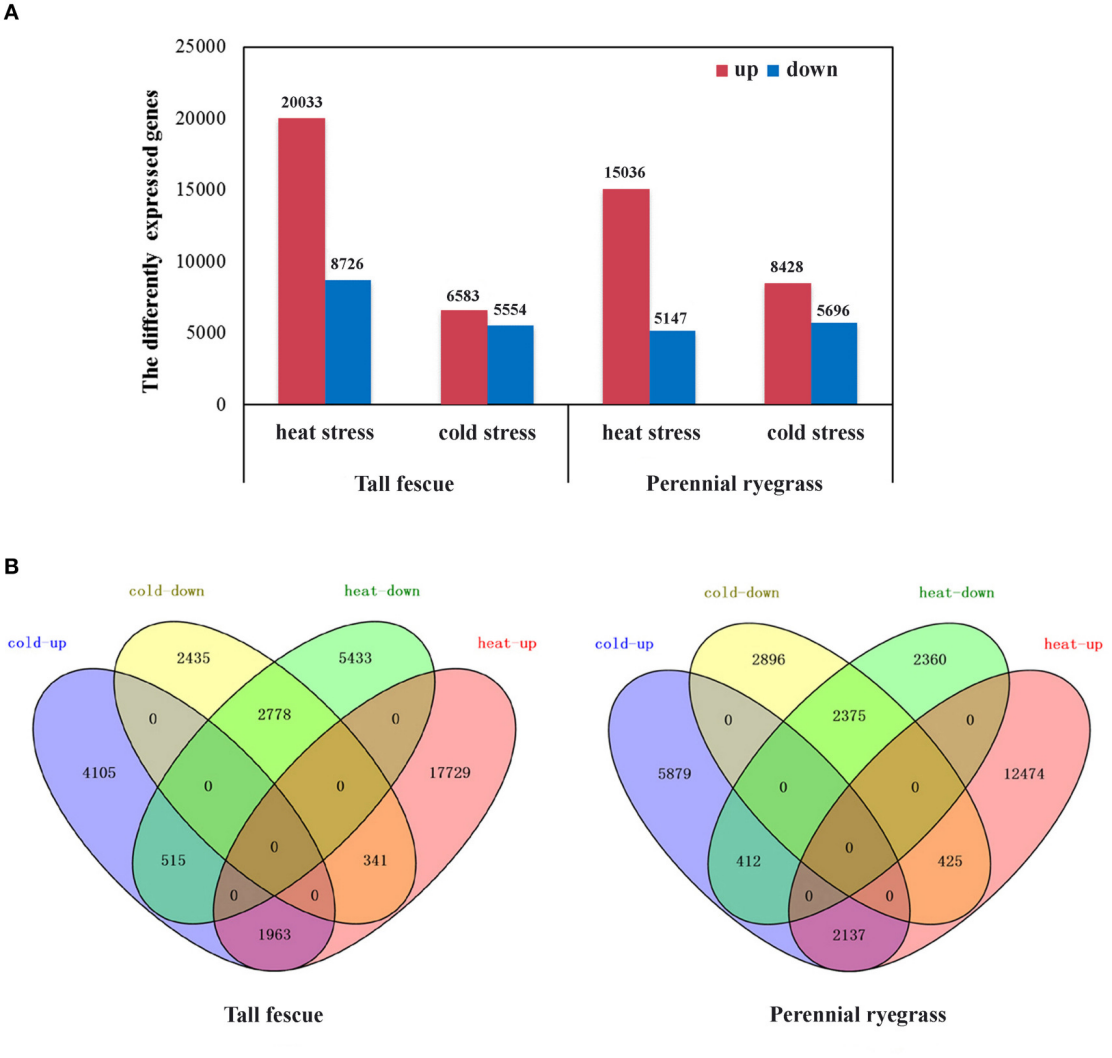
**Analysis of differentially expressed genes (DEGs) in tall fescue and perennial ryegrass transcriptomes in response to heat and cold stress. (A)** Up- and down-regulated DEGs under heat or cold stress in the two grass species. Up (red) indicates upregulated DEGs, and down (blue) represents downregulated DEGs. **(B)** Shared and divergent DEGs in the tall fescue or perennial ryegrass under heat and cold stress. Heat-up (red) indicates the upregulated DEGs under heat stress; heat-down (green) represents the downregulated DEGs under heat stress; cold-up (blue) denotes the upregulated DEGs under cold stress; and cold-down (yellow) represents the downregulated DEGs under cold stress.

We next compared expression level of genes in the transcriptome to identify differentially expressed genes (DEGs) in tall fescue and perennial rye grass under temperature stress. DEGs were judged by the thresholds of “FDR < 0.001 and |log_2_Ratio| ≥ 1. Tall fescue under heat stress (HS) had 28,759 (29.48% of all the unigenes) DEGs, where 20,033 of the DEGs were upregulated and 8726 DEGs were downregulated. Tall fescue under cold-stress (CS) showed a total of 12 137 DEGs (12.44% of all unigenes), which consisted of 6583 upregulated DEGs and 5554 downregulated DEGs (Figure 1A). In both stress conditions, a total of 5597 DEGs showed overlapping differences in expression, including the following: (1) 1963 co-upregulated DEGs, (2) 2778 co-downregulated DEGs, (3) 341 DEGs upregulated in HS but downregulated in CS, and (4) 515 DEGs upregulated in CS but downregulated in HS (Figure 1B). The DEGs common between HS and CS accounted for 19.5% of the heat-DEGs and 46.1% of the cold-DEGs.

In perennial ryegrass under HS, we detected a total of 20,183 DEGs (27.60% of all unigenes), including 15,036 upregulated and 5147 downregulated DEGs. Under CS, we detected a total of 14,124 DEGs (19.31% of all unigenes), with 8428 upregulated DEGs and 5696 downregulated DEGs (Figure 1A). Similar to our results in tall fescue, we detected 5349 overlapping DEGs under both stresses, which were expressed as follows: (1) 2137 co-upregulated, (2) 2375 co-downregulated, (3) 425 upregulated in HS but downregulated in CS, and (4) 412 upregulated in CS but downregulated in HS (Figure 1B). In perennial rye grass, common DEGs accounted for 26.5% of the heat-DEGs and 37.9% of the cold-DEGs.

In both species, GO-term analysis of the DEG-response to HS was enriched (*P* < 0.05) in various biological process categories, such as “response to heat,” “photosynthesis,” “response to oxidative stress,” and “response to inorganic substance,” whereas the DEG-response to CS was enriched (*P* < 0.05) in “oxidation-reduction process,” “response to inorganic substance,” and “response to cold” (Supplementary Tables 1-S3, 2-S3). In addition, the common DEGs under both stresses were transcription factors, starch synthases, protein kinases, heat shock proteins, cytochrome P450s, and several unidentified proteins. Interestingly, we observed a very strinking difference in expression of *Hsf* genes in both stresses. We focused our further analyses on the *Hsf* genes to better understand how both species respond to temperature stress.

### Classification of Hsf proteins of the two grass species

**Figure 2:**
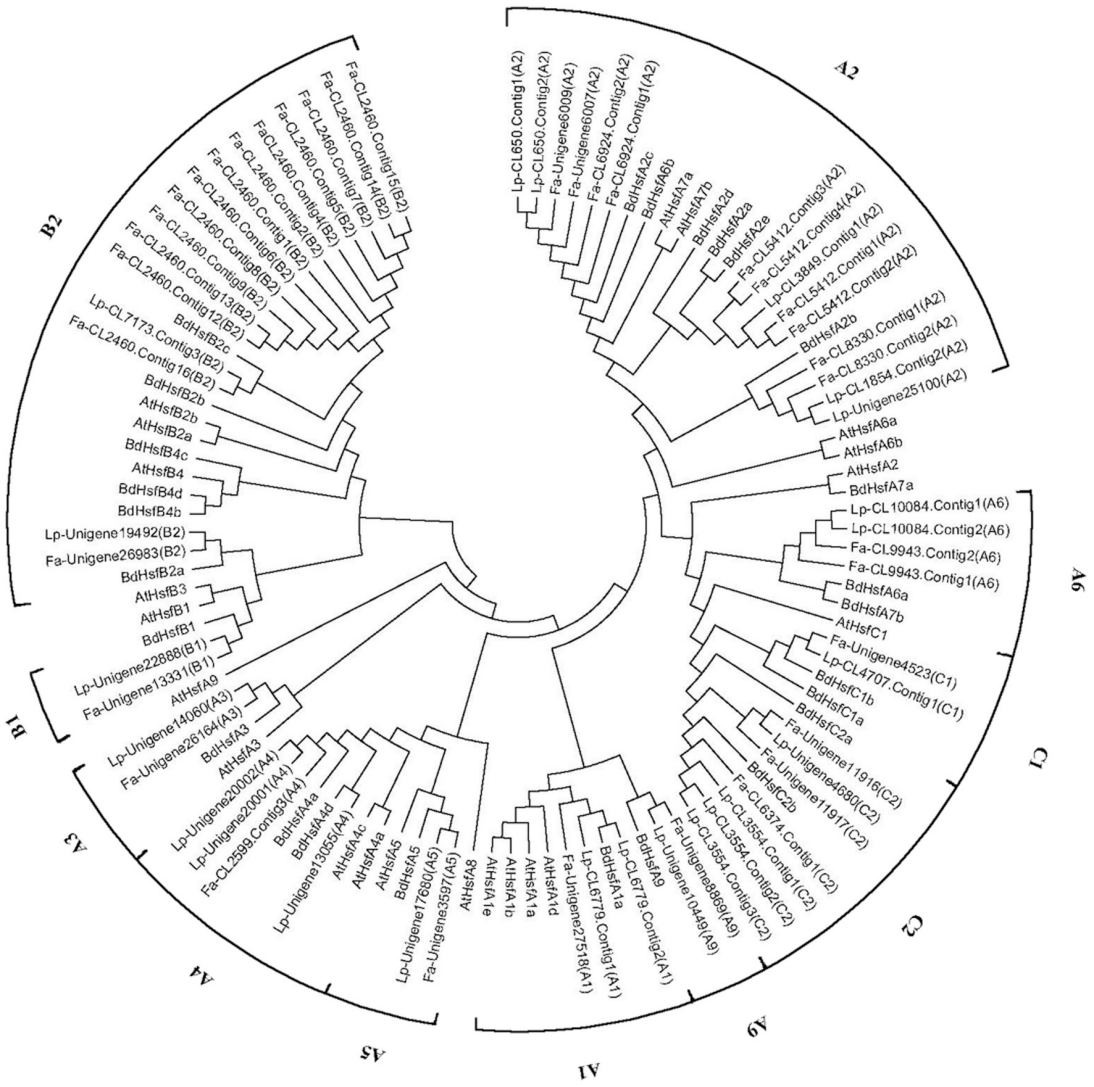
**Neighbor–joining phylogenetic tree of Hsf proteins from the tall fescue, perennial ryegrass, ***Brachypodium distachyon***, and ***Arabidopsis thaliana*****. Hsfs in *Brachypodium distachyon* are prefixed by Bd, and Hsfs in *Arabidopsis thaliana* are prefixed by At. Hsfs in tall fescue are marked by a “Fa” prefix and sequence-ID in the transcriptome. Hsfs in perennial ryegrass are marked by a “Lp” prefix and a sequence-ID in the transcriptome.

Hsf proteins have been categorized into different subfamilies and classes in model plant species such as *A. thaliana*, tomato, and *B. distachyon* (Scharf et al., 2012). Based on the characteristic sequences of *Hsf* genes identified in these model plant species, we identified 74 and 52 unigenes as *Hsfs* in the tall fescue and perennial ryegrass transcriptome, respectively. To better understand the potential function and homology of Hsf genes in both grass species, we conducted a phylogenetic analysis of Hsf proteins from the tall fescue, perennial ryegrass, *B. distachyon*, and *A. thaliana* (Figure 2). One of the main characteristics of Hsf proteins is the DNA-binding domain. In our *de novo* transcriptome assembly, we recovered some potential *Hsf* mRNA sequences that did not contain the characteristic Hsf DNA-bidning domain. These sequences were excluded from the phylogenetic analysis.

Hsf proteins are categorized into subfamilies HsfA, HsfB, and HsfC. Our phylogenetic analysis uncovered seven smaller clusters within subfamily HsfA, whereas subfamilies HsfB and HsfC formed distinct groups. The same Hsf subfamily members for tall fescue and perennial ryegrass showed high sequence similarity. Hsfs from the two grass species were more closely related to *B.distachyon* Hsfs than *A. thaliana* Hsfs. In tall fescue and perennial ryegrass, we recovered seven HsfA classes (A1, A2, A3, A4, A5, A6, and A9), two HsfB classes (B1 and B2), and two HsfC classes (C1 and C2). Both grass species contained a large number of A2 and B2 class members, and we also detected C2 members that are unique in monocot species. However, we did not detect sequences belonging to subclasses A7, A8, B3, and B4 in the two grass species (Supplementary Tables 1-S4, 2-S4).

### Expression profile of Hsf family genes

**Figure 3:**
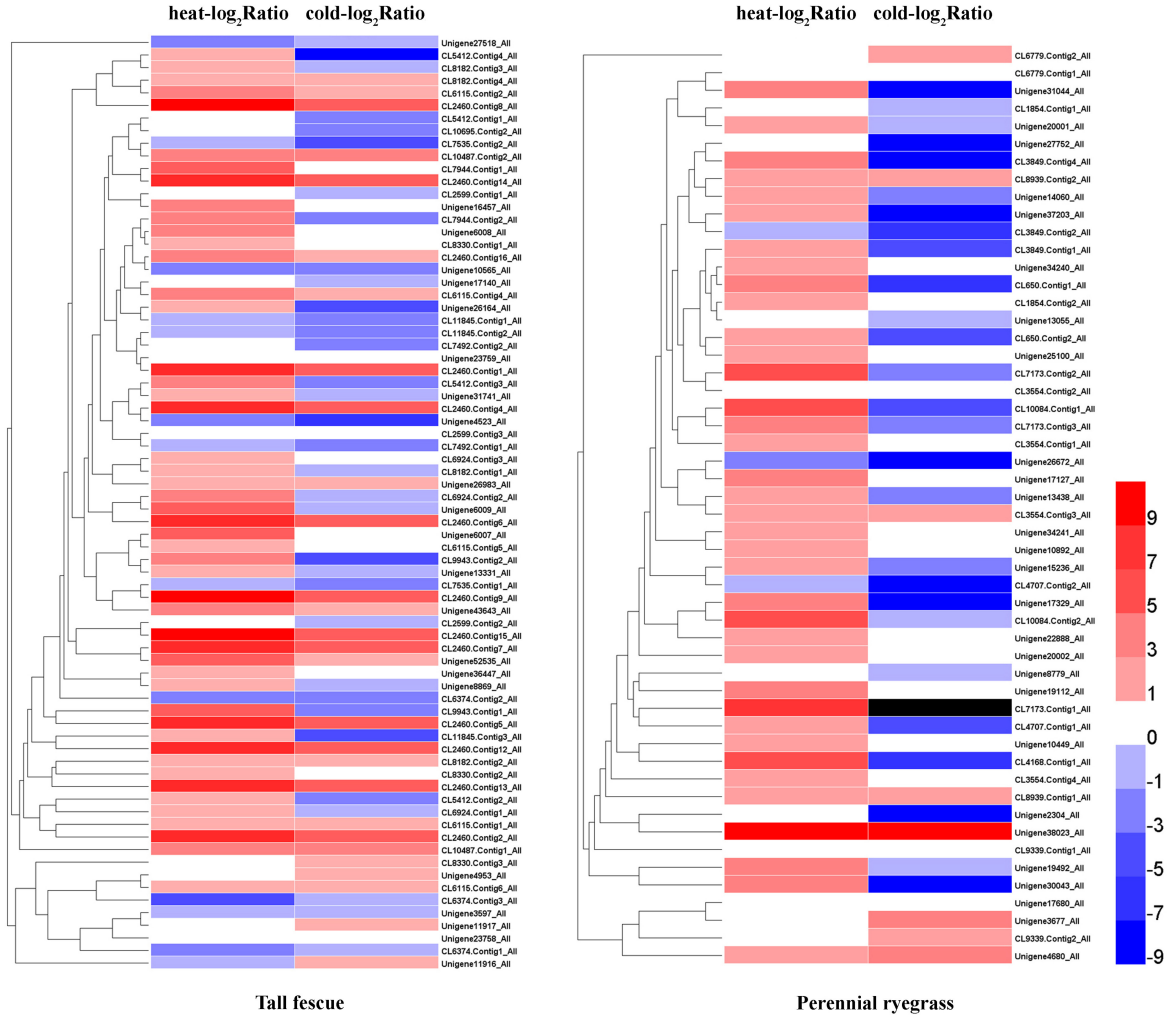
**Heatmaps of the expression profiles of ***Hsf*** genes in the two grass species subjected to heat and cold stress**. Heat-log_2_ Ratio indicates the fold-change of *Hsf* FPKM values under HS, and cold-log_2_ Ratio represents the fold-change of *Hsf* FPKM values under CS. The black grid denotes genes where we did not detect expression.

**Figure 4:**
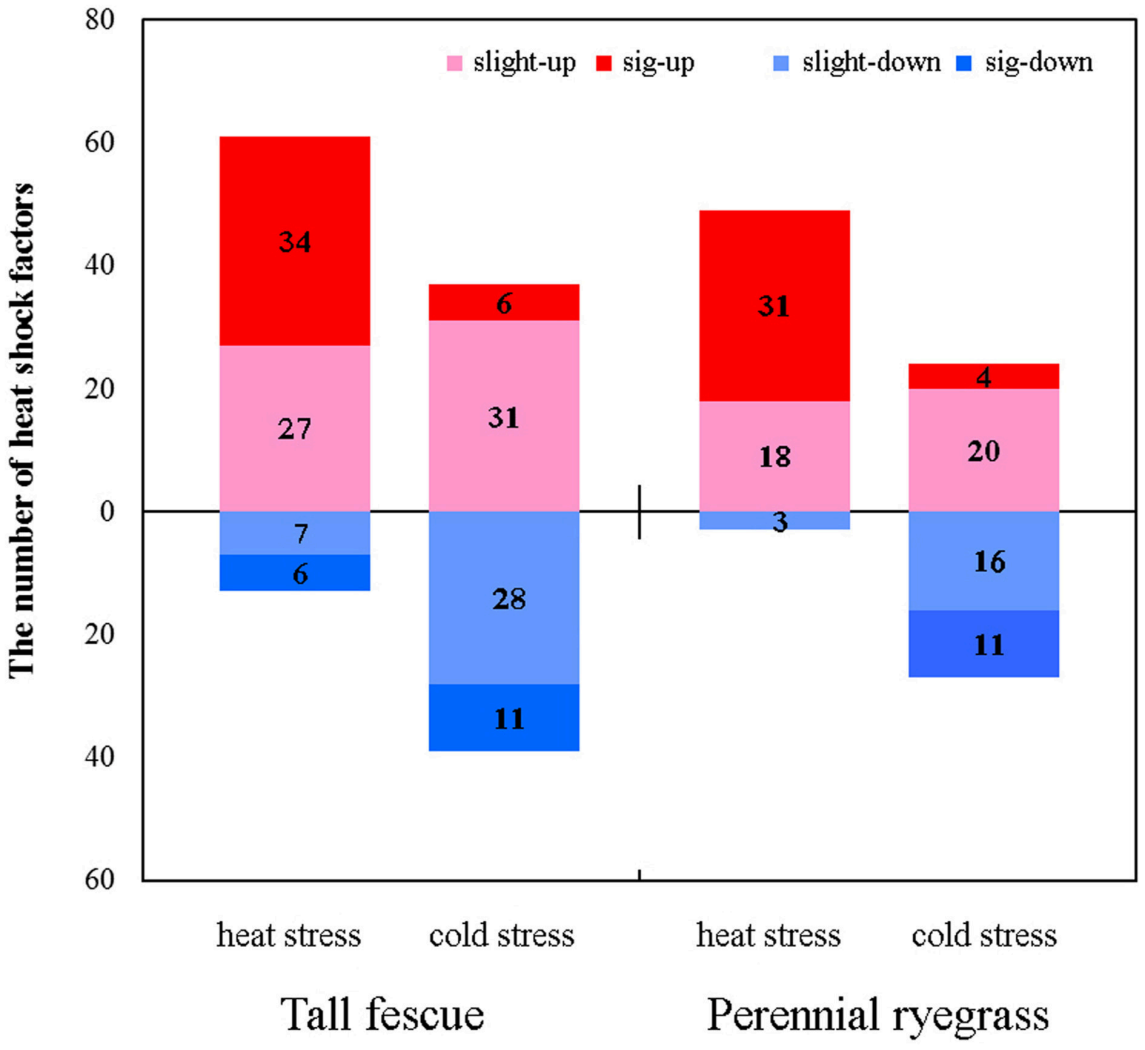
**The expression patterns of heat shock factors in the tall fescue and perennial ryegrass under heat and cold stress**. Slight-up (pink) indicates insignificantly upregulated *Hsf* genes, sig-up (red) represents significantly upregulated *Hsf* genes, slight-down (light blue) denotes insignificantly downregulated *Hsf* genes, and sig-down (blue) denotes significantly downregulated *Hsf* genes. Significant differences in transcript expression levels were determined by setting the thresholds for false discovery rate as “(FDR) < 0.001” and “|log_2_ Ratio| ≥ 1,” where ratio indicates the fold-change of FPKM values for the treatment and control samples.

In both grass species, HS resulted in higher number of up-regulated *Hsf* genes compared to CS (Figure 3). In tall fescue, HS resulted in upregulation of 61 *Hsfs* including 34 significantly differentially expressed *Hsfs* (DE-*Hsfs*) and downregulation of 13 *Hsfs* (6 DE-*Hsfs*), whereas CS resulted in 37 upregulated *Hsfs* (6 DE-*Hsfs*) and 39 downregulated *Hsfs* (11 DE-*Hsfs*) (Figure 3). The expression differences of *Hsf* genes were more pronounced in perennial ryegrass, where HS resulted in 49 upregulated *Hsfs* (31 DE-*Hsfs*) and 3 downregulated *Hsfs* (no DE-*Hsfs*), and CS resulted in 24 upregulated *Hsfs* (4 DE-*Hsfs*) and 27 downregulated *Hsfs* (11 DE-*Hsfs*) (Figure 4). In addition, one *Hsf* (CL7173.Contig1_All) in perennial ryegrass was not expressed under CS, but was slightly upregulated under HS (Figure 4).

**Figure 5:**
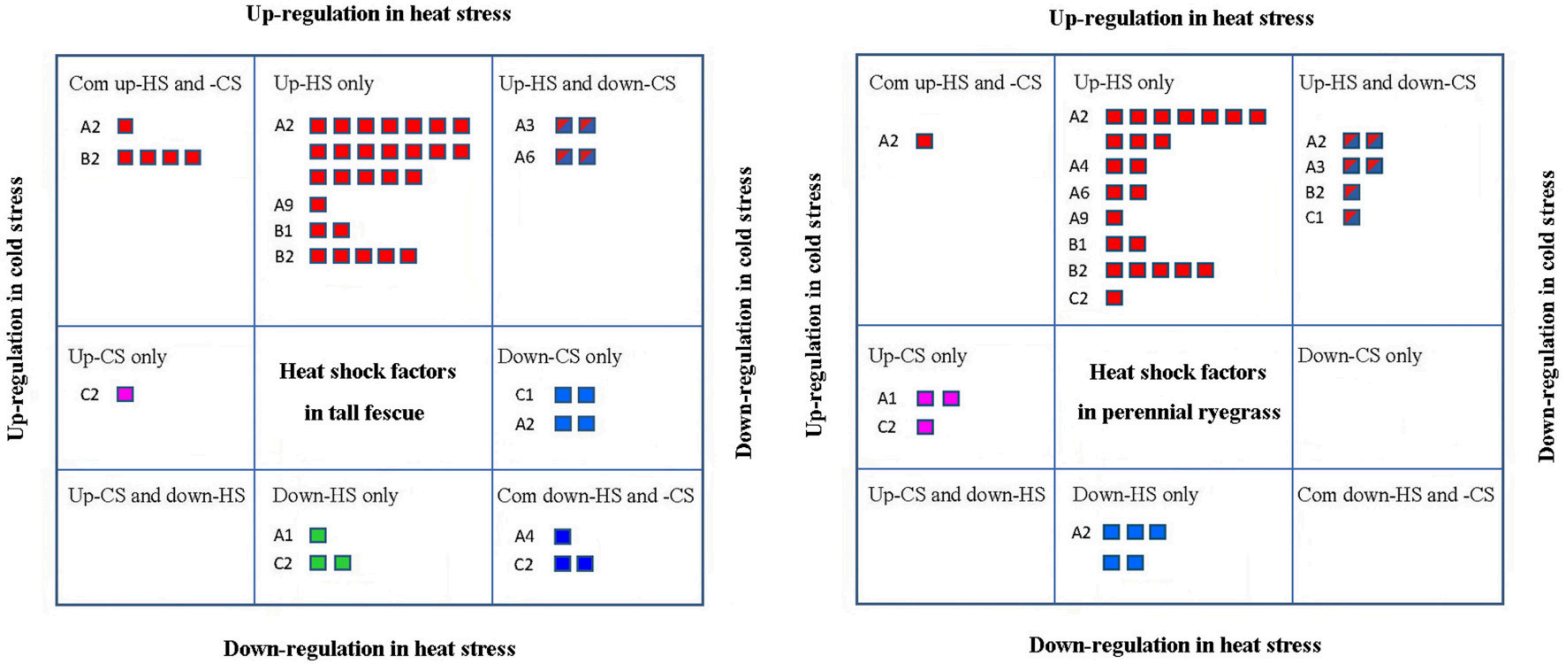
**Stress-response analyses of different ***Hsf*** classes showing significant expression differences at least one stress condition**. “Up-HS only” indicates upregulated heat shock factors (*Hsfs*) only under heat stress (HS); “Down-HS only” represents downregulated *Hsfs* only under HS; “Up-CS only” represents upregulated *Hsfs* only under cold stress (CS); and “Down-CS only” denotes downregulated *Hsfs* only under CS. “Com up-HS and CS” indicates upregulated *Hsfs* under HS and CS, and “Com down-HS and CS” represents downregulated *Hsfs* under HS and CS. “Up-HS and Down-CS” shows *Hsfs* upregulated under HS and downregulated under CS, and “Up-CS and Down-HS” denotes *Hsfs* downregualted under HS and upregulated under CS.

Next, we assessed the responses of different class *Hsf* genes to heat and cold stresses in the two grass species. *Hsfs* that showed significant differential expression under at least one stress conditions were classified and listed in Figure 5. In tall fescue, there were 25 *Hsfs* that showed significantly increases only in HS-treatment, including classes A2, A9, B1, and B2, and in perennial ryegrass, 24 *Hsfs* showed HS-specific upregulation, including *Hsf* A2, A4, A6, A9, B1, B2, and C2 classes. Of these, the classes *Hsf* A2 and B2 in the tall fescue and *Hsf* A2 in perennial ryegrass were significantly upregulated under HS and CS stresses. Members of classes *Hsf* A2 and A4 were significantly downregulated in CS in both grass species. Class *Hsf* A3 in both species and *Hsf* A6 in tall fescue were significantly upregulated in HS but downregulated in CS. In tall fescue, some *Hsf* C2 class genes showed significant upregulation only under CS, whereas other *Hsf* C2 class genes showed downregulation under both temperature stresses. In contrast, perennial ryegrass *Hsf* C2 class genes were upregulated by both temperature stresses, and downregulated only under CS (Figure 5).

### Analysis of Hsf target genes in the transcriptomes of the two grass species

HSP genes are one of the main targets of Hsf proteins under stress conditions. In the transcriptomes, we detected 371 transcripts encoding *HSP* genes in tall fescue, and 347 transcripts in perennial ryegrass (Table 1). These *HSPs* represented all five families. In both species and stress conditions, the strongest response was observed in the Hsp20 and Hsp70 families, then by the Hsp90 and Hsp60 families. The weakest response was detected in the Hsp100 family (Supplementary Tables 1-S5, 2-S5). HS resulted in significant upregulation of 199 and 192 *HSPs* in tall fescue and perennial ryegrass. HS also showed significant downregulation of 34 and 23 *HSPs* in tall fescue and perennial ryegrass, respectively. CS resulted in significant upregulation of 69 and 45 *HSPs*, and downregulation of 24 and 45 *HSPs* in tall fescue and perennial ryegrass, respectively (Table 1).

**Table 1:** **Genes encoding Heat shock proteins (HSPs), ascorbate peroxidases (APXs), inositol-3-phosphate synthases (IPSes), and four galactinol synthases (GOLSes) that show expression level changes responding to heat and cold stress in tall fescue and perennial ryegrass**.

		**Gene sum**	**Heat stress**		**Cold stress**	
			**Upregulated**	**Downregulated**	**Upregulated**	**Downregulated**
Tall fescue	*HSP*	371	199	34	69	24
	*APX*	36	7	0	0	1
	*IPS*	0	0	0	0	0
	*GOLS*	16	2	9	6	0
Perennial ryegrass	*HSP*	347	192	23	45	45
	*APX*	25	5	4	1	5
	*IPS*	5	3	0	0	0
	*GOLS*	5	0	0	3	0

**Figure 6:**
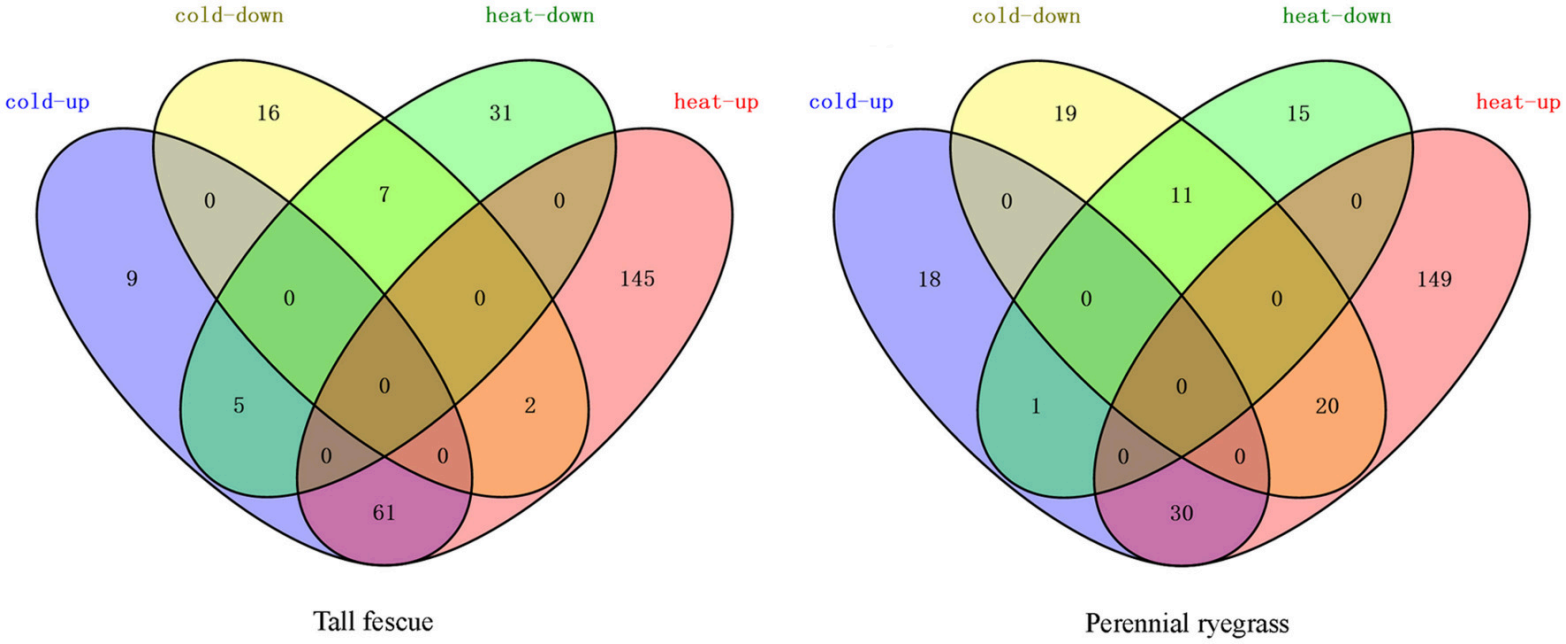
**Venn diagram of differentially expressed target genes of ***Hsfs*** in the tall fescue or perennial ryegrass under heat and cold temperature stress**. Red (heat-up) represents the upregulated target transcripts under heat stress; green (heat-down) represents the downregulated target transcripts under heat stress; blue (cold-up) indicates the upregulated target transcripts under cold stress; and yellow (cold-down) represents the down-regulated target transcripts under cold stress.

We next analyzed other known Hsf targets in the transcriptome. In tall fescue, we detected 36 *APXs*, 16 *GOLS* genes, but did not detect any *IPS* genes. In perennial ryegrass, we detected 25 *APXs*, five *IPSes*, and five *GOLSes* (Table 1, Supplementary Tables 1-S6, 2-S6). HS resulted in significant increase in the expression of *APXs* in both grass species, and *IPSes* in perennial ryegrass. In contrast, CS resulted in a significant increase in the expression of *GOLSes* in two species (Table 1). The Venn diagram in Figure 6 shows the distribution of shared and divergent target genes under two temperature stresses in each grass species.

### Changes in the expression levels of Hsfs and target genes

**Figure 7:**
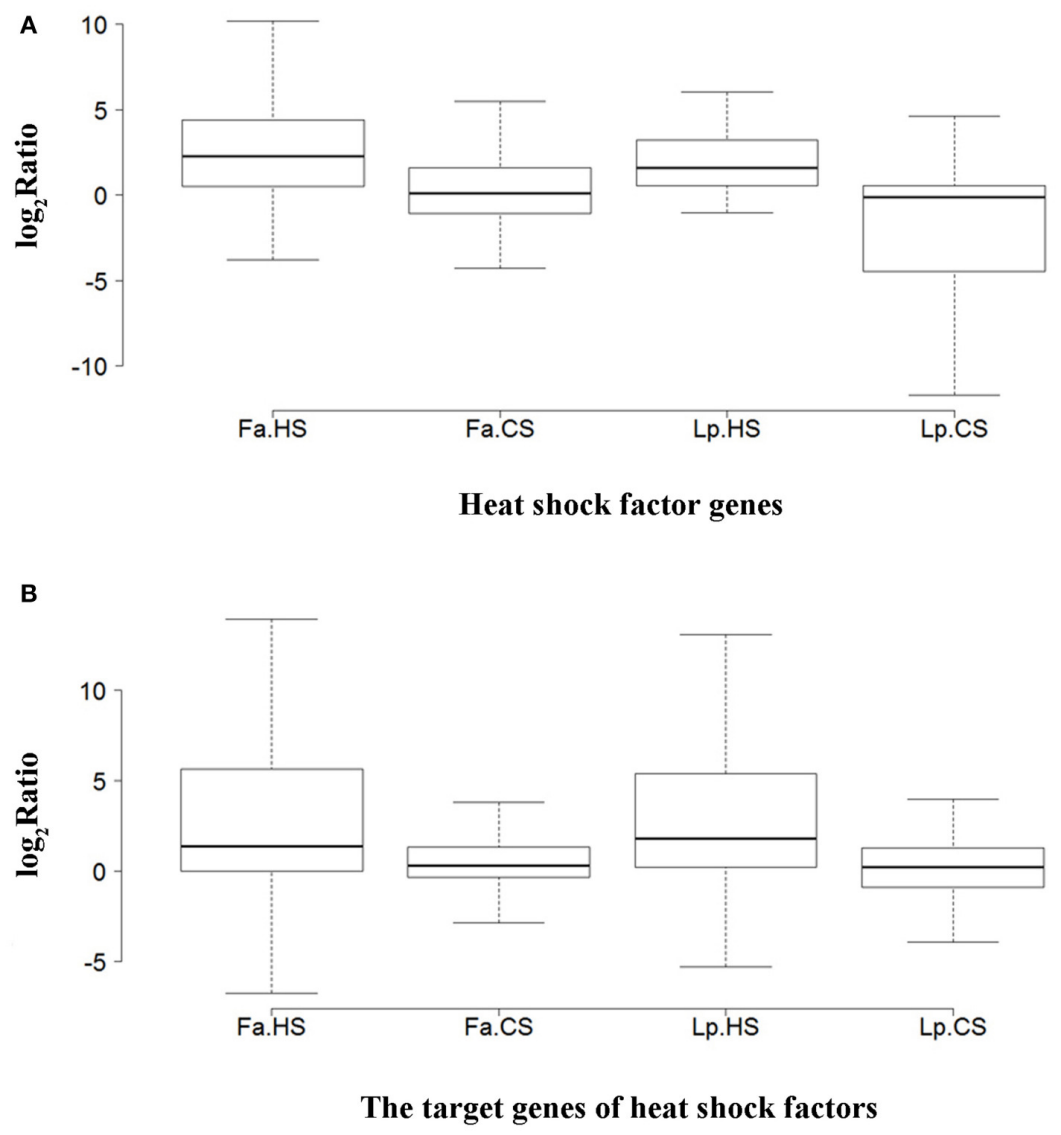
**Overall expression differences (log_**2**_ Ratio) of heat shock factors and their target genes between species and stresses**. **(A)** Heat shock factor genes. **(B)** The target genes of heat shock factors. Abbreviations are as follows: Fa, tall fescue (*Festuca arundinacea*); Lp, perennial ryegrass (*Lolium perenne*); HS, heat stress; and CS, cold stress. The log_2_ Ratio represents the fold-change of *Hsf* FPKM values under heat or cold treatment relative to that of the control samples.

To understand the difference of expression profiles between species and stress responses, we analyzed the expression of *Hsfs* and their target genes under different temperatures in tall fescue and perennial ryegrass. *Hsfs* were expressed at low levels in the control, where only three *Hsfs* were detected to be >10 FPKM in tall fescue, with a peak FPKM value of 40.80, and two *Hsfs* in perennial ryegrass, with highest FPKM level of 12.76. Under HS, more than 30% of the *Hsfs* in the two grass species were expressed at >10 FPKM, and the highest FPKM value was 102.75 in the tall fescue and 104.25 in perennial ryegrass. In contrast, under CS, seven *Hsfs* showed >10 FPKM in tall fescue, with a peak FPKM value of 30.72, and one *Hsf* in perennial ryegrass with a 10.62 FPKM value. Comparison changes in *Hsf* expression levels in HS and CS, we observed that HS had a greater effect on *Hsfs* in the tall fescue relative to perennial ryegrass. The median log_2_Ratio in the tall fescue *Hsfs* was slightly higher than that observed in perennial ryegrass under the two temperature stresses (Figure 7A).

We next analyzed the expression changes of *Hsf* target genes, including *HSP, APX, IPS*, and *GOLS* genes. Under the control temperature, the highest level of *HSP* expression was 431.15 FPKM in the tall fescue and 453.01 FPKM in perennial ryegrass. Under HS, the highest FPKM of *HSPs* in the tall fescue and perennial ryegrass was 4436.84 and 3586.88, respectively. Under CS, the highest FPKM value was 458.05 and 210.59, respectively. For *APXs*, the peak value of FPKM under control, HS and CS was 383.27, 827.92, and 472.44 in tall fescue, and 277.42, 267.16, and 314.56 in perennial ryegrass, respectively. For *IPSes*, these peak values were 10.53, 27.03, and 156.44 in tall fescue, respectively, and 8.96, 9.83, and 17.39 in perennial ryegrass. For *GOLSes*, they were 33.63, 9.12, and 38.51 in tall fescue, and 10.12, 8.96, and 22.90 in perennial ryegrass, respectively. The expression levels of Hsf target genes did not differ between the two grass species, whereas differences were observed between the two temperature stresses conditions (Figure 7B).

### qRT-PCR validation of differentially expressed transcripts from RNA-seq

To confirm the accuracy and reproducibility of the transcriptome results, 64 unigenes (32 from each grass transcriptome) were randomly chosen for quantitative real-time -PCR (qRT-PCR). These included several *Hsfs* and other genes. The primer sequences, FPKM, and qRT-PCR values are listed in Supplementary Table 3. The qPCR results showed a general agreement (75–78%) with the transcript abundance as determined by RNA-Seq. Specifically, we observed an agreement of 75% in the tall fescue and 78% in perennial ryegrass, which suggest that the RNA-Seq data was a reliable read-out for differential expression (Supplementary Table 3).

## Discussion

### Comparative analysis of transcriptomes of the tall fescue and perennial ryegrass under different temperature stresses

In subtropical regions, the growth of grass is limited by high temperatures in the summer and low temperatures during winter. However, heat and cold stress are not generally lethal because plants have a variety of protective mechanisms to tolerate temperature changes by altering expression of stress-response genes (Krasensky and Jonak, 2012). The leaves of plants, being exposed to air, generally receive the stress signals first, and rapidly respond to these environmental changes. We therefore used leaf samples of the tall fescue and perennial ryegrass under heat, cold, and normal temperatures for deep RNA-sequencing on an Illumina HiSeq 2000 platform. After *de novo* assembly, 97,565 and 73,125 unigenes were generated in the tall fescue and perennial ryegrass transcriptomes. Of these, about two-thirds were functionally annotated using various reference databases. In the transcriptome of heat-tolerant and heat-sensitive cultivars of tall fescue responding to high-temperature stress, 31,803 unigenes were assembled (Hu et al., 2014). In a transcriptome of perennial ryegrass generated by 454 sequencing, 9399 non-redundant contigs were acquired from 25,744 high-quality EST reads after *de novo* assembly (Studer et al., 2012). By using the RNA-seq strategy, *de novo* assembly of the perennial ryegrass transcriptome resulted in 185,833 transcripts, in which over 50,000 transcripts were functionally annotated (Farrell et al., 2014). Comparing our transcriptome to previously published ones suggest that the data obtained in our whole-genome-wide transcriptional sequencing were larger and sufficient in size for analyzing differentially expressed genes and mining for temperature-resistance genes in the two species.

In our study, approximately 30% and 25% of all genes in the transcriptome were identified as DEGs (differentially expressed genes) during heat or cold stress, with a greater number of DEGs showing upregulation than downregulation. HS exhibited a higher number of DEGs than CS, which may be attributed to the fact that the two grass species being more heat-tolerance. In addition, being exposed to below-freezing temperatures can result in to lethal mechanical damages to the plant, such as the formation of ice crystals within cells (intracellular freezing) and between cell walls (extracellular freezing) (Xin and Browse, 2000). We may have recovered a low number of gene expression changes in the below-freezing temperature conditions due to the mechanical damage that occurred before the plant could induce the cold acclimation gene-expression response. This suggests that sudden freezing stress can result in more severe injury to the plants compared to that observed during HS.

### Heat shock factors in response to heat or cold stress

Transcription factors are one of the master regulators that lead to changes in the level of expression of downstream genes, thereby influencing plant metabolism (Ramirez and Basu, 2009). In the present study, the expression the transcription factor gene family *Hsf* were remarkably impacted by heat and cold stresses. A high proportion of *Hsfs* showed significant upregulation upon HS, whereas some *Hsfs* were upregulated by CS. Recent studies involving various plant models show that *Hsfs* respond not only to HS, but also to other abiotic stimuli such as waterlogging, salt, osmosis, and oxidative stresses (Ogawa et al., 2007; Yoshida et al., 2008; Shim et al., 2009; Liu et al., 2011; Xue et al., 2014). We conducted a transcriptome analysis of two non-model species in response to temperature stresses, and mainly focused on *Hsfs* and their target genes.

We identified 74 *Hsfs* in the tall fescue and 52 *Hsfs* in perennial ryegrass transcriptomes. The numbers of *Hsf* genes in model organisms are 21 in *A. thaliana*, 24 in *B. distachyon*, 25 in rice, 30 in poplar (*Populus trichocarpa*), 40 in *Gossypium raimondii*, and 56 in wheat (Scharf et al., 2012; Xue et al., 2014); therefore the *Hsf* gene family in the tall fescue was larger than those in most known species. The three plant *Hsf* subfamilies, *Hsf* A, *Hsf* B, and *Hsf* C were detected in the tall fescue and perennial ryegrass, and we assigned each gene to A1 to A6 and A9, B1 and B2, C1 and C2 classes. All subfamilies/classes are represented in monocots and eudicots, except for C2, which is exclusive to monocots (Scharf et al., 2012). We uncovered most members in the subfamily *Hsf* A, which was in agreement with known plant *Hsf* families. However, classes A7, A8, and B4, which are widely distributed in most plant species, were not detected in the two grass species. At present, *Hsf* A9 genes are considered to be confined to eudicots and play a special role in seed development (Almoguera et al., 2002; Kotak et al., 2007; Carranco et al., 2010). Nevertheless, we retrieved one *Hsf* A9 gene in tall fescue and two in perennial ryegrass. *Hsf* A9 genes were induced by HS, suggesting that *Hsf* A9 may also be related to heat tolerance, but potentially not involved in cold tolerance. Overall, general patterns were similar to model species, but we recovered some distinct differences in *Hsf* members between grasses and other monocots or eudicot species.

Analysis of *Hsfs* showing significant differences in gene expression in both grass species exposed to HS or CS suggested that *Hsfs* responded more strongly to heat. Among all of the *Hsf* classes, the members of class *Hsf* A2 were highest in quantity, and most of the *Hsf* A2 genes were upregulated under HS. A related study in tomato, *A. thaliana*, and rice also showed that *Hsf* A2 genes were most strongly induced upon exposure to long-term HS or repeated cycles of HS and recovery (Charng et al., 2007; von Koskull-Doring et al., 2007; Chan-Schaminet et al., 2009; Nishizawa-Yokoi et al., 2011; Liu and Charng, 2013). These studies suggested that *HsfA2* improves thermotolerance based not only on expression abundance during heat-stress, but also based on the formation of the superactivator complex HsfA1/A2, which has a much higher level of activation for HSP-encoding genes than single Hsf proteins. However, in our HS analysis, *Hsf* A1 in perennial ryegrass was weakly upregulated, and was downregulated in the tall fescue. These results may suggest that *Hsf A2* tall fescue and perennial ryegrass independently function to enhance thermotolerance. With a few exceptions, the *Hsf* A2d gene, which is a member of class *Hsf* A2, was upregulated during CS. To date, direct evidence on how *Hsf* A2d improves cold tolerance remains elusive. *A. thaliana Hsf* A2 knockout lines and *Hsf* A2-overexpression lines indicate that *Hsf* A2 is one of the key regulators in protecting organelles against oxidative damage (Miller and Mittler, 2006; Zhang et al., 2009). Plant stress often results in the overproduction of reactive oxygen species; therefore *Hsf* A2 may be involved in improving cold tolerance by alleviating oxidative damage.

Likewise, HS also resulted in a significant increase in the expressions of *Hsf* A3, A4, and A6 in the two grasses, which was consistent to results observed in wheat under HS (Xue et al., 2014). It has been suggested that these *Hsf* genes participate in the process of acquiring thermotolerance. In *Arabidopsis* undergoing heat and drough stress, Hsf A3 is involved in the transcriptional cascade that is downstream of the DREB2A stress-regulatory system (Chen et al., 2010). Interestingly, we observed a significant upregulation of *DREB* transcripts in the two grass species. It is therefore possible that *Hsf* A3 and *DREB* may interact in these two species, although this requires further testing. *HsfA4a* in *A. thaliana* was regulated by the mitogen-activated protein kinases MPK3 and MPK6 to confer enhanced tolerance to salt stress (Pérez-Salamó et al., 2014). *HsfA4a* in wheat and rice confers Cd tolerance by upregulating the gene expression of *metallothionein* (Shim et al., 2009). *A. thaliana HsfA6a* responds strongly to exogenous ABA, NaCl, and drought, and acts as a transcriptional activator for stress-responsive genes via the ABA-dependent signaling pathway (Hwang et al., 2014). In our transcriptome analysis, we observe a HS-induced response in most members of subfamily *HsfA* in the two grasses. Although *HsfA* genes are currently the most extensively studied in the *Hsf* family, the function and mechanism of all of its members still remain unclear.

*Hsf* subfamily B classes B1 and B2 in the tall fescue and perennial ryegrass were upregulated under HS, whereas *Hsf* B2 genes in tall fescue were upregulated under CS. Based on the protein domains present in *Hsf* subfamily B genes, it is generally suggested that subfamily B does not have transcription activator function (von Koskull-Doring et al., 2007). However, tomato HsfB1 shows strong synergistic activation by cooperating with Hsf subfamily A proteins or other transcriptional activators (Bharti et al., 2004). *A. thaliana* HsfB1 and HsfB2b also appear to be necessary for the expression of HS-inducible *HSP* genes (Ikeda et al., 2011; Pick et al., 2012). *HsfB2b* in *A. thaliana* represses the expression of PRR7 and sustains circadian rhythm during heat and salt stress, which are important functions for plant growth and fitness (Kolmos et al., 2014). *Arabidopsis HsfB2a* is involved in gametophyte development, and a heat-inducible long non-coding antisense RNA controls its expression (Wunderlich et al., 2014). Our results supports the hypothesis that *Hsf* B subfamily genes are involved in temperature stress-response.

*Hsf* subfamily C genes were particularly enhanced in tall fescue and perennial ryegrass undergoing CS. To date, the function of *Hsf* subfamily C genes remain elusive. Our results suggested that *Hsf* C genes may be involved in cold tolerance. Interestingly, similar results have been reported in wheat and rice. An activation motif in the C-terminal domain of the wheat *TaHsfC2a* contains an activation motif in the C-terminal domain, and regulates the expression of *HSP* genes (Xue et al., 2014). Of the 26 rice *Hsfs*, 10 showed a response to low temperature stress (Mittal et al., 2009).

### Targets genes of Hsfs in response to heat and cold stresses

The expression of *Hsfs* triggers changes in expression of downstream target genes. One of the major targets of Hsfs is the *HSP* genes, and HSPs play a role in enhancing thermotolerance in various plants (Timperio et al., 2008; Tripp et al., 2009). Several studies have also shown that HSPs can improve cold tolerance. For example, Hsp70 serves as a molecular chaperone at low temperature *in vitro* (Zhang and Guy, 2006). DcHsp17.7 from carrot can prevent cold-induced protein degradation, and confer cold tolerance (Song and Ahn, 2010). HSPs expression levels change under various stressors, which indicate similarities in plant adaption mechanisms in response to abiotic stresses (Wang et al., 2004; Krasensky and Jonak, 2012). Here, we found that *HSPs* show strong response to HS and CS in the tall fescue and perennial ryegrass, indicating that they may be involved in protecting cells against temperature stresses.

Other target genes of Hsfs include *APX, IPS*, and *GOLS*. It has been suggested that Hsfs act as molecular sensors that directly sense ROS signals, which in turn activate the expression of *HSPs* and oxidative stress response genes. Hsf-binding motifs have been detected in the promoter region of genes involved in the ROS gene network, including defense genes, H_2_O_2_-scavenging enzyme APX, and related transcription factors (Miller and Mittler, 2006). Three stress-related genes in *A. thaliana*, (*APX2, IPS2*, and *GOLS1*) are regulated by HsfA2 (Miller and Mittler, 2006; Scharf et al., 2012). Here, we specifically investigated the expression levels of these three genes under HS and CS. The expression of *APXs* and *IPSes* significantly increased upon HS, and *GOLSes* decreased under HS, but increased under CS. These results suggest that there may be a crosstalk between *Hsfs* and stress genes during ROS signaling.

### Difference between two stresses and similarity of two species

Most DEGs were different between HS and CS in the tall fescue or perennial ryegrass, and a small portion of DEGs were commonly expressed under the two stress conditions. A previous report involving *A. thaliana* indicated the existence of a greater crosstalk between drought and high-salinity stress signaling processes compared to those involved in cold and high-salinity (Matsui et al., 2008). Transcriptome response of *Zea mays* to various adverse environments show a greater gene overlap between heat and salt stress compared to those induced by heat and cold (Fernandes et al., 2008). The results of the present study are in agreement to these findings, and show that genes responding to HS and CS show differences, irrespective of species. However, the DEGs were classified under the same GO category. Each GO class or gene family included many genes with functional divergence. Consequently, it is possible that different members in the same gene family or functional cluster respond to stressors differently. Some genes can also be induced by several different stresses. The difference in how *Hsf* family and their targets genes respond to different temperature treatments in the present study support this hypothesis.

Overall, the gene expression profiles of tall fescue and perennial ryegrass showed a similar response to the temperature stress. In terms of *Hsfs* and their target genes in the two grasses, their responses were almost identical in the same stress conditions. Previous reports have considered that the tall fescue has a greater heat tolerance compared to the perennial ryegrass (Raeside et al., 2012). However, in a previous study, we did not detect any physiological differences in how these plants responded to thermotolerance (Wang et al., 2014). In the present study, the two grasses showed similar changes in gene expression when subjected to HS or CS. The response mechanism of the two grasses to temperature may therefore be similar. Therefore, our results suggest that there are less differences between the two grass species, and more differences between the two temperature stresses.

## Conclusions

By using RNA-seq analysis, we compared the transcriptomes of tall fescue and perennial ryegrass under HS and CS. We generated a total of 97,565 unigenes in the tall fescue and 73,125 unigenes in the perennial ryegrass through *de novo* transcriptome assembly. In addition, 28,759 and 12,137 significant DEGs under HS and CS were obtained in the tall fescue, and 20,183 and 14,124 in the perennial ryegrass, respectively. In each species, HS resulted in more DEGs than that observed under CS, and less overlap was detected under the two temperature conditions. However, DEGs in the two grasses exposed to the same stress showed similar GO-term functions. Using sequence similarity and phylogenetic analysis, we identified 74 *Hsf* members in the tall fescue and 52 *Hsfs* in the perennial ryegrass, and assigned these to three subfamilies, HsfA, HsfB, and HsfC. This included seven HsfA classes, two HsfB classes, and two HsfCs classes. *Hsf* expression was significantly affected by HS and CS, with a larger enhancement observed under HS. We recovered the highest number of members in subfamily *Hsf* A, and classes A2, A3, A4, A6, and A9 genes were upregulated by HS. Class *HsfA2* was quite expansive in the two species, which showed a greater change of expression level under HS. Accordingly, *HsfA2* members may be key regulators of HS response in these species. Subfamily *HsfB* was upregulated by both HS and CS, and the expression of *HsfC* genes were mainly upregulated under CS. These results suggested that subfamily *HsfA* are likely involved in acclimating the two grasses to heat tolerance, and *HsfB* plays a role in adaptation to the both stresses, whereas subfamily *HsfC* is related to the acquirement of cold tolerance. We also analyzed the expression profiles of the target genes of Hsfs. HSPs, the major target genes of Hsfs, were significantly upregulated under the two stresses, suggesting that HSPs may play important roles in protecting plant cells against the high and low temperature damage. For other target genes of Hsfs, the expression of *APX* and *IPS* in both grasses were significantly enhanced by HS, whereas that of the *GOLS* genes increased under CS. Similar to the *Hsfs*, different members of these target genes showed specific response to HS and/or CS. The results from the two species serve as useful references to investigate how other grass species respond to temperature-stress. Further, studies will clarify the function of potential candidate *Hsf* members, and identify the gene regulation network involving *Hsfs* under HS or CS conditions.

## Author contributions

YW and XM designed the experimental plan and drafted and revised the manuscript. YD, JW, and HY participated in preparing, treating, and collecting samples. XT, HC, and YW analyzed and interpreted the sequence data. All authors read and approved the final manuscript.

### Conflict of interest statement

The authors declare that the research was conducted in the absence of any commercial or financial relationships that could be construed as a potential conflict of interest.

## Supplementary material

Supplementary Figure 1 **Gene Ontology (GO) annotation of transcripts in (A) tall fescue and (B) perennial ryegrass**.

Supplementary Figure 2 **Functional classification using Clusters of Orthologous Groups of proteins (COG) in tall fescue (A) and perennial ryegrass (B)**.

Supplementary Table 1 **Annotation of tall fescue transcriptome in response to heat and cold stress**.

- Supplementary Table 1-S1: Expression levels and functional annotation of transcripts of the tall fescue transcriptome.
- Supplementary Table 1-S2: KEGG pathway mapping of the tall fescue transcriptome.
- Supplementary Table 1-S3: GO-Biological Process enrichment (*p* < 0.05) of the differentially-expressed genes (DEGs) showing significant expression differences in tall fescue under heat and cold stress.
- Supplementary Table 1-S4: Genes encoding heat shock factor (*Hsf*) genes in the tall fescue transcriptome.
- Supplementary Table 1-S5: Genes encoding heat shock protein (*HSP*) genes in the tall fescue transcriptome.
- Supplementary Table 1-S6: Genes encoding Ascorbate peroxidase (*APX*), Inositol-3-phosphate synthase (*IPS*) and Galactinol synthase (*GOLS1*) in the tall fescue transcriptome.

Supplementary Table 2 **The annotation of perennial ryegrass transcriptome in response to heat and cold stress**.

- Supplementary Table 2-S1: Expression levels and functional annotation of transcripts in the perennial ryegrass transcriptome.
- Supplementary Table 2-S2: KEGG pathway mapping of the perennial ryegrass transcriptome.
- Supplementary Table 2-S3: GO-Biological Process enrichment (*p* < 0.05) of differentially-expressed genes (DEGs) showing significant expression differences in perennial ryegrass under heat and cold stress.
- Supplementary Table 2-S4: Genes encoding heat shock factor (*Hsf*) genes in the perennial ryegrass transcriptome.
- Supplementary Table 2-S5: Genes encoding heat shock protein (*HSP*) genes in the perennial ryegrass transcriptome.
- Supplementary Table 2-S6: Genes encoding Ascorbate peroxidase (*APX*), Inositol-3-phosphate synthase (*IPS*) and Galactinol synthase (*GOLS1*) in the perennial ryegrass transcriptome.

Supplementary Table 3 **Comparison of gene expression patterns between RNA-seq and quantitative real-time PCR results**.
