# Supplementary figures and images for: Heat Shock Factor Genes of Tall Fescue and Perennial Ryegrass in Response to Temperature Stress by RNA-Seq Analysis
Source: Front Plant Sci. 2016 Jan 11;6:1226. doi: 10.3389/fpls.2015.01226 (PMC4707269; doi:10.3389/fpls.2015.01226)

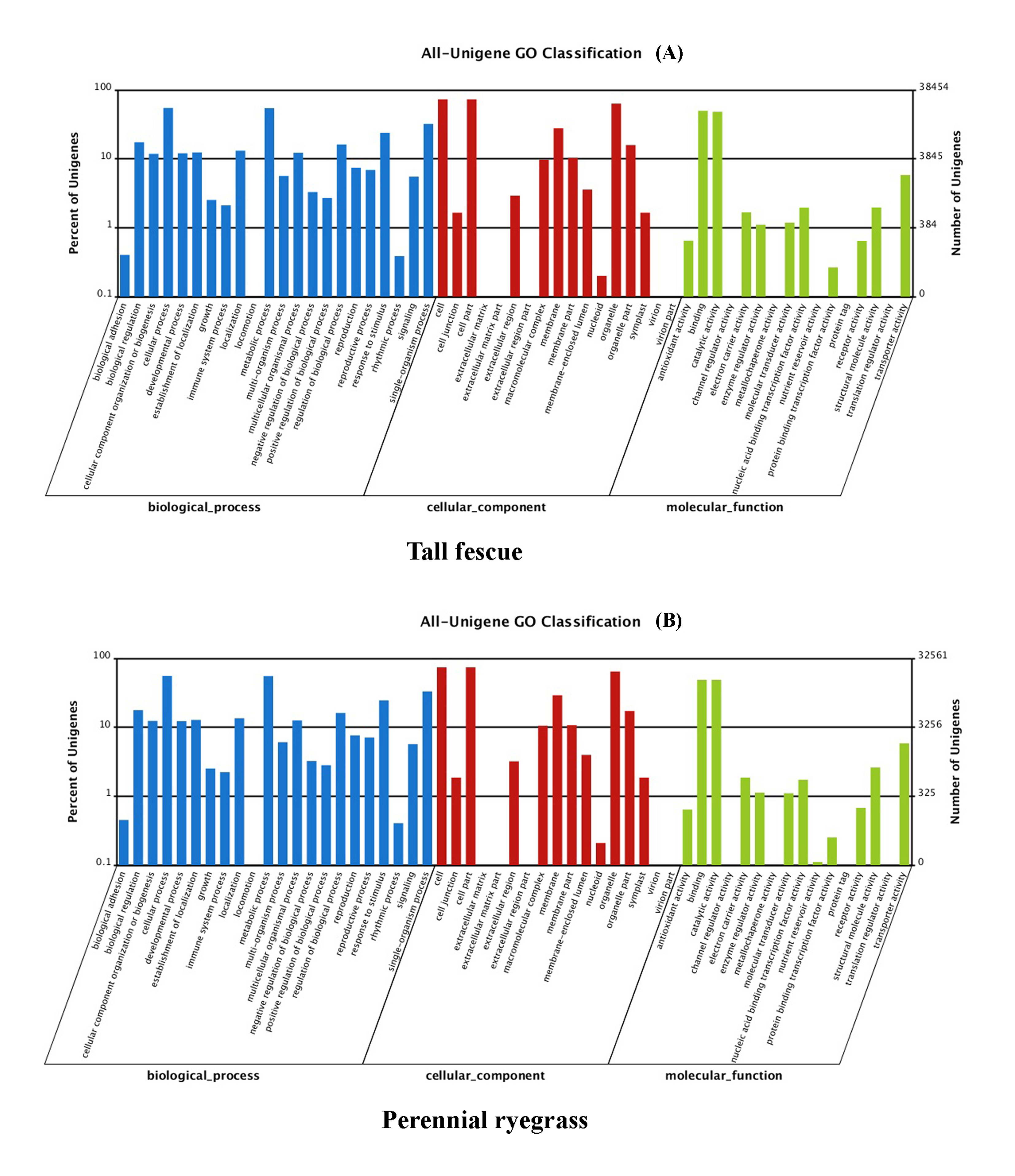

Supplement: Supplementary Figure 1 — Gene Ontology (GO) annotation of transcripts in (A) tall fescue and (B) perennial ryegrass. [file Image1.jpg]

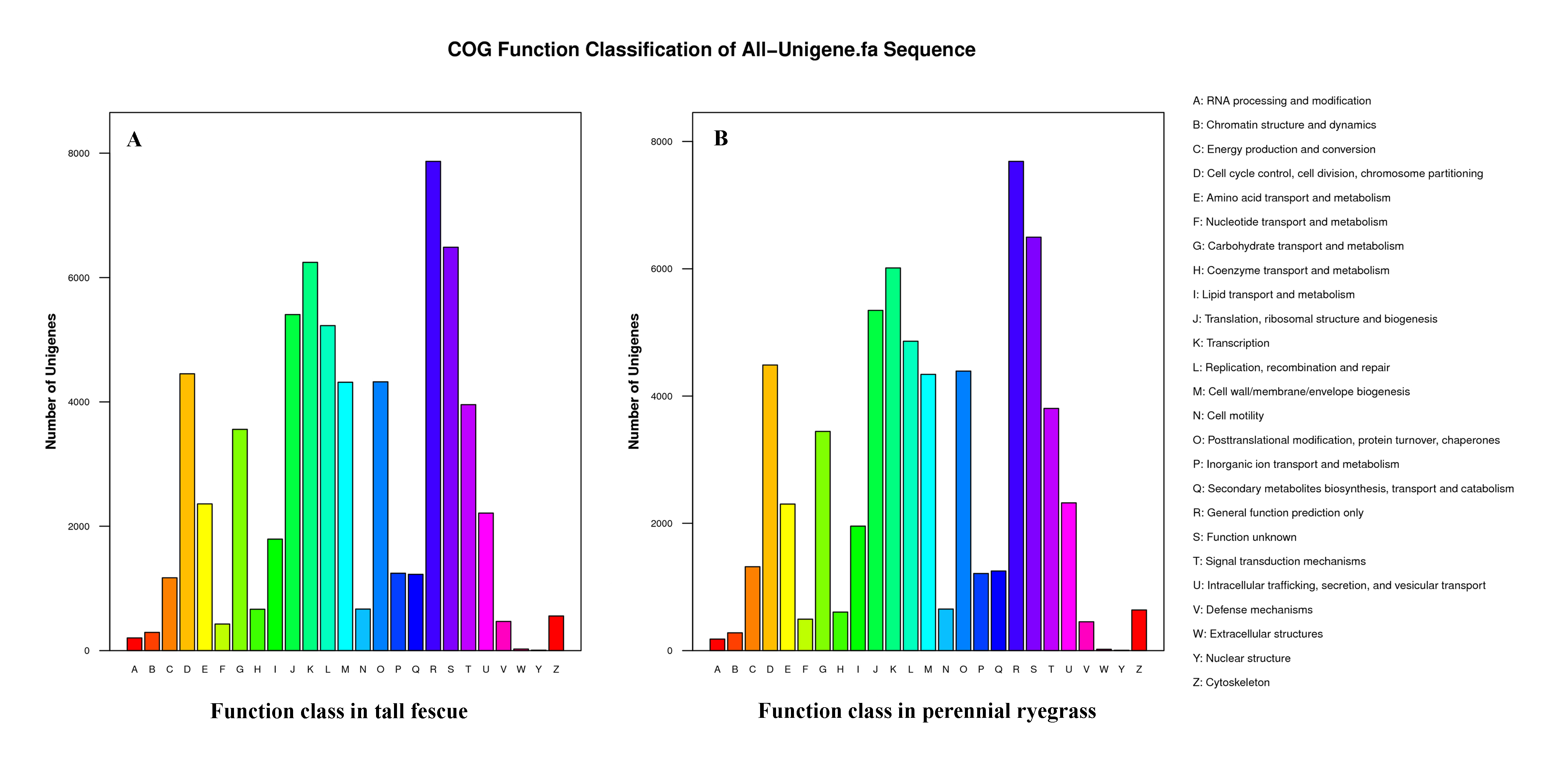

Supplement: Supplementary Figure 2 — Functional classification using Clusters of Orthologous Groups of proteins (COG) in tall fescue (A) and perennial ryegrass (B). [file Image2.jpg]
